# Radiomics Analysis of Iodine-Based Material Decomposition Images With Dual-Energy Computed Tomography Imaging for Preoperatively Predicting Microsatellite Instability Status in Colorectal Cancer

**DOI:** 10.3389/fonc.2019.01250

**Published:** 2019-11-22

**Authors:** Jingjun Wu, Qinhe Zhang, Ying Zhao, Yijun Liu, Anliang Chen, Xin Li, Tingfan Wu, Jianying Li, Yan Guo, Ailian Liu

**Affiliations:** ^1^Department of Radiology, First Affiliated Hospital, Dalian Medical University, Dalian, China; ^2^Translational Medicine Team, GE Healthcare (China), Shanghai, China; ^3^GE Healthcare (China), Shanghai, China

**Keywords:** microsatellite instability, colorectal neoplasms, iodine-based material decomposition image, radiomics, dual-energy computed tomography

## Abstract

**Purpose:** The aim of this study was to investigate the value of radiomics analysis of iodine-based material decomposition (MD) images with dual-energy computed tomography (DECT) imaging for preoperatively predicting microsatellite instability (MSI) status in colorectal cancer (CRC).

**Methods:** This study included 102 CRC patients proved by postoperative pathology, and their MSI status was confirmed by immunohistochemistry staining. All patients underwent preoperative DECT imaging scanned on either a Revolution CT or Discovery CT 750HD scanner, and the iodine-based MD images in the venous phase were reconstructed. The clinical, CT-reported, and radiomics features were obtained and analyzed. Data from the Revolution CT scanner were used to establish a radiomics model to predict MSI status (70% samples were randomly selected as the training set, and the remaining samples were used to validate); and data from the Discovery CT 750HD scanner were used to test the radiomics model. The stable radiomics features with both inter-user and intra-user stability were selected for the next analysis. The feature dimension reduction was performed by using Student's *t*-test or Mann–Whitney *U*-test, Spearman's rank correlation test, min–max standardization, one-hot encoding, and least absolute shrinkage and selection operator selection method. The multiparameter logistic regression model was established to predict MSI status. The model performances were evaluated: The discrimination performance was accessed by receiver operating characteristic (ROC) curve analysis; the calibration performance was tested by calibration curve accompanied by Hosmer–Lemeshow test; the clinical utility was assessed by decision curve analysis.

**Results:** Nine top-ranked features were finally selected to construct the radiomics model. In the training set, the area under the ROC curve (AUC) was 0.961 (accuracy: 0.875; sensitivity: 1.000; specificity: 0.812); in the validation set, the AUC was 0.918 (accuracy: 0.875; sensitivity: 0.875; specificity: 0.857). In the testing set, the diagnostic performance was slightly lower with AUC of 0.875 (accuracy: 0.788; sensitivity: 0.909; specificity: 0.727). A nomogram based on clinical factors and radiomics score was generated via the proposed logistic regression model. Good calibration and clinical utility were observed using the calibration and decision curve analyses, respectively.

**Conclusion:** Radiomics analysis of iodine-based MD images with DECT imaging holds great potential to predict MSI status in CRC patients.

## Introduction

Colorectal cancer (CRC) is the third most common cancer and the second leading cause of cancer-related death worldwide (1). The occurrence and development of CRC are accompanied by a series of genetic abnormalities, of which microsatellite instability (MSI) is an important pathway in carcinogenesis (2). According to previous reports, even though MSI occurs in only approximately 15% of CRCs, it has gained considerable attention by clinicians owing to its significant value for CRC prognosis and treatment (2, 3). Microsatellite stability (MSS) status is maintained by the mismatch repair (MMR) genes, which are applied to repair genetic sequences that have been erroneous during replication in normal tissues. When MMR system is impaired, the error microsatellite sequences will accumulate, resulting in MSI and early onset of CRC (2). Obtaining MSI status is necessary because the MSI CRC tissues possess special biological behaviors, they are more likely to have a better prognosis and benefit from immunotherapy, and they may be resistant to fluorouracil chemotherapy (4). However, the methods for assessing MSI status including immunohistochemistry (IHC) and polymerase chain reaction (PCR) are all based on pathological tissues obtained by invasive methods. And these advanced biological tests have not been widely generalized owing to the limitation of advanced medical equipment in local institutions (5). Thus, development of non-invasive and cost-effective method for predicting MSI status could be meaningful for clinicians to obtain more diagnostic clues and guide further treatment strategies.

Given the growing number of applications in clinical diagnosis, dual-energy computed tomography (DECT) has been considered as a milestone in CT imaging because it can provide quantitative measurements to characterize the lesions (6). DECT can generate accurate iodine-based material decomposition (MD) images, which can reflect the vascularization of various tissues via measuring the contrast material (iodine) concentration (IC) (7–9). And the correlation between IC values and MSI status has been reported in previous studies (10, 11). However, from the iodine-based MD images, we can only routinely obtain the mean value of IC in lesions, and more imaging characteristics such as heterogeneity remain untapped. Radiomics analysis achieved the conversion of medical images to high-dimensional mineable data to quantitatively and comprehensively describe tissues' characteristics from imaging (12). Several scholars have reported that the radiomics features extracted from CT images showed some value in predicting MSI status in CRC patients; however, the diagnostic performance was limited (13, 14). Accordingly, we have presumed that the radiomics analysis of iodine-based MD images might serve as a non-invasive and reproducible way to preoperatively assess MSI status in CRC patients and set up a study to investigate its diagnostic efficacy.

## Materials and Methods

### Patient Population

**Figure 1:**
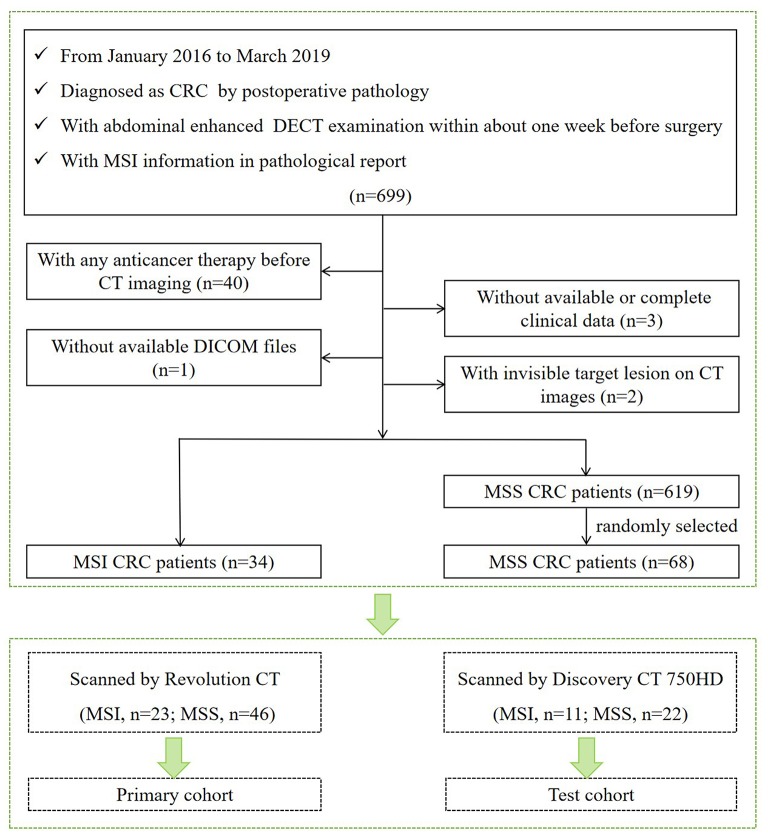
Flowchart of patient selection process.

Our institutional review board approved this retrospective study with waiver of the informed consent. Patients examined in our institution from January 2016 to March 2019 who met the following criteria were included in our study. Inclusion criteria are as follows: (1) underwent curative-intent surgical resection and diagnosed as CRC by postoperative pathology; (2) underwent abdominal enhanced DECT examination within about 1 week before surgery; and (3) with MSI information tested by IHC staining in pathological report. Exclusion criteria are (1) with any local or systematic anticancer therapy (radiotherapy, chemotherapy, and biotherapy) before CT imaging; (2) without available digital imaging data and communications in medicine (DICOM) files in our system; (3) without available or complete clinical data; and (4) with invisible target lesion on CT images. According to the outcomes of MSI testing in the pathological report, we collected 653 CRC patients including 34 MSI CRC patients (incidence rate of 5.2%) and 619 MSS CRC patients. For further statistical analysis, 34 MSI CRC patients (23 scanned on Revolution CT and 11 scanned on Discovery CT 750HD) and 68 controls with MSS CRC (46 scanned on Revolution CT and 22 scanned on Discovery CT 750HD) in a 1:2 ratio (15) (randomly selected from 619 MSS CRC patients) were ultimately included in our study (61 males and 41 females; age: 63.82 ± 11.51 years; range 26–87 years). The flowchart of patient selection process is shown in Figure 1. The demographics of CRC patients is listed in Table 1. The clinical data of all CRC patients including age, gender, carcinoembryonic antigen (CEA) (normal level, 0–5 U/ml), carbohydrate antigen 19-9 (CA19-9) (normal level, 0–27 U/ml), alcohol history, smoking history, hypertension history, diabetes history, and family history of cancer were recorded. The included CRC patients were divided into two independent cohorts: (1) primary cohort: CRC patients examined on the Revolution CT scanner were used to establish a radiomics model to predict MSI status (70% samples were randomly selected as the training set, and the remaining samples were used to validate); and (2) testing cohort: CRC patients examined on the Discovery CT 750HD scanner were used to test the predictive model.

**Table 1:** Demographics of CRC patients.

**Characteristic**	**Primary cohort (Revolution CT) (*****n*** **=** **69)**		**Validation cohort (Discovery CT 750HD) (*****n*** **=** **33)**	
	**MSI (*n* = 23)**	**MSS (*n* = 46)**	**MSI (*n* = 11)**	**MSS (*n* = 22)**
Age (years) (mean ± SD)	60.22 ± 11.95	63.5 ± 10.96	61.18 ± 10.83	69.59 ± 11.01
**Gender**, ***n*** **(%)**				
Male	12 (52.2)	28 (60.9)	7 (63.6)	14 (63.6)
Female	11 (47.8)	18 (39.1)	4 (36.4)	8 (36.4)
**CEA**, ***n*** **(%)**				
Normal	17 (73.9)	31 (67.4)	7 (63.6)	9 (40.9)
Abnormal	6 (26.1)	15 (32.6)	4 (36.4)	13 (59.1)
**CA19-9**, ***n*** **(%)**				
Normal	19 (82.6)	39 (84.8)	9 (81.8)	15 (68.2)
Abnormal	4 (17.4)	7 (15.2)	2 (18.2)	7 (31.8)
**Alcohol history**, ***n*** **(%)**				
Yes	2 (8.7)	4 (8.7)	2 (18.2)	2 (9.1)
No	21 (91.3)	42 (91.3)	9 (81.8)	20 (90.9)
**Smoking history**, ***n*** **(%)**				
Yes	1 (4.3)	8 (17.4)	2 (18.2)	2 (9.1)
No	22 (95.7)	38 (82.6)	9 (81.8)	20 (90.9)
**Hypertension**, ***n*** **(%)**				
Yes	7 (30.4)	21 (45.7)	1 (9.1)	5 (22.7)
No	16 (69.6)	25 (54.3)	10 (90.9)	17 (77.3)
**Diabetes**, ***n*** **(%)**				
Yes	3 (13)	8 (17.4)	1(9.1)	1 (4.5)
No	20 (87)	38 (82.6)	10 (90.9)	21 (95.5)
**Family history of cancer**, ***n*** **(%)**				
Yes	2 (8.7)	1 (2.2)	2 (18.2)	0 (0)
No	21 (91.3)	45 (97.8)	9 (81.8)	22 (100)
CT-reported tumor size (cm) (mean ± SD)	2.46 ± 1.41	1.83 ± 1.39	2.83 ± 1.76	2.11 ± 1.61
**CT-reported tumor location**, ***n*** **(%)**				
Right colon	12 (52.2)	14 (30.4)	7 (63.6)	13 (59.1)
Left colon	9 (39.1)	26 (56.5)	2 (18.2)	6 (27.3)
Rectum	2 (8.7)	6 (13)	2 (18.2)	3 (13.6)
**CT-reported serous invasion**, ***n*** **(%)**				
Yes	21 (91.3)	28 (60.9)	8 (72.7)	18 (81.8)
No	2 (8.7)	18 (39.1)	3 (27.3)	4 (18.2)
**CT-reported lymph node invasion**, ***n*** **(%)**				
Yes	17 (73.9)	32 (69.6)	8 (72.7)	9 (40.9)
No	6 (26.1)	14 (30.4)	3 (27.3)	13 (59.1)

*CA19-9, carbohydrate antigen 19-9; CEA, carcinoembryonic antigen; CRC, colorectal cancer; MSI, microsatellite instability; MSS, microsatellite stability*.

### Microsatellite Instability Status Assessment

The MSI status was assessed by IHC staining of MMR proteins (MLH1, MSH2, PMS2, and MSH6). IHC staining was routinely performed based on postoperative tissues via standard streptavidin biotin-peroxidase procedure. According to the staining results of MMR proteins, patients were classified into the MSI or MSS group. CRC tissues with at least one of four negatively stained MMR proteins were defined as MSI CRC; others with four positively stained proteins were defined as MSS CRC (2).

### Iodine-Based Material Decomposition Image Acquisition and Analysis

The abdominal DECT scans were performed on a Revolution CT scanner or Discovery CT 750HD scanner (GE Healthcare) in supine position. The non-enhanced abdominal CT scan was performed first with the conventional CT protocol of using the tube voltage of 120 kVp. The contrast-enhanced CT scans were performed using the dual-energy spectral CT scanning mode using the following scan parameters: helical, rapid switch between tube voltages of 80 and 140 kVp in 0.5 ms; tube current, 230–445 mA; detector width, 80 mm; helical pitch, 0.992:1 on the Revolution CT scanner and 1.375:1 on the Discovery CT 750HD scanner; rotation time, 0.6–0.8 s; slice thickness, 1.25 mm; and slice interval, 1.25 mm. For the contrast-enhanced CT scans, 1.2 ml/kg of non-ionic contrast media iohexol (Omnipaque 300 mg/ml, GE Healthcare) was used. The contrast medium was administered via the antecubital vein at an injection rate of 3 ml/s. The arterial phase, venous phase, and delayed phase scans were obtained after 30, 60, and 120 s following the administration of contrast agents. The CT scans covered the abdomen and pelvis from the dome of diaphragm to pubic symphysis. After CT scans, the iodine-based MD images in the venous phase were reconstructed at 1.25-mm image slice thickness and interval using the Gemstone Spectral Imaging (GSI) software on an advanced workstation 4.6 (AW 4.6; GE Healthcare).

Image analysis was performed by an abdominal radiologist with 3 years of experience and independently verified by another trained radiologist with 5 years of experience to reduce possible bias. Their discrepant interpretations were resolved via consultation. These observers were blinded to all clinical and pathological information of CRC patients. The following data extracted from CT images were analyzed and recorded: (a) tumor size, defined as the maximum axial diameter of tumors on images; (b) tumor location, subclassified as right colon, left colon, and rectum; (c) CT-reported serous invasion, defined as irregular projections from the serosal surface, and/or clouding of the pericolic fat, and/or loss of the normal fat planes, and/or thickened contiguous fascial reflections; (d) CT-reported lymph node invasion, defined as enlarged lymph node (short-axis diameter > 1 cm), and/or clustered at least three lymph nodes (16).

### Tumor Segmentation and Radiomics Feature Extraction

**Figure 2:**
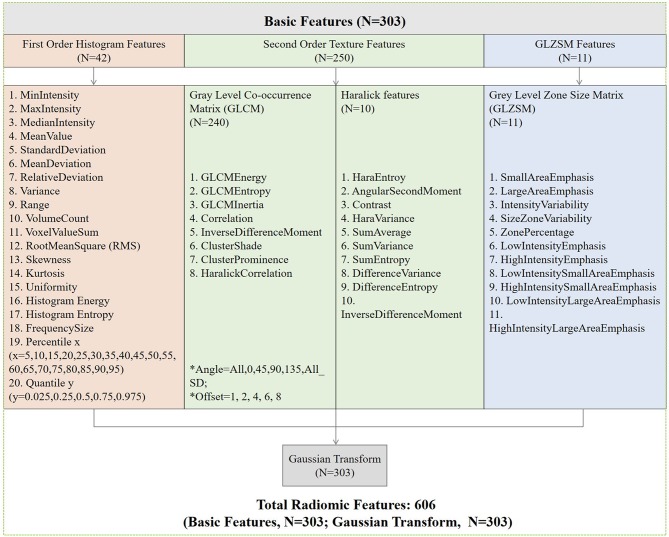
Details of radiomics features: (1) first-order histogram features (*n* = 42); (2) second-order texture features: gray level co-occurrence matrix (GLCM) (*n* = 240), Haralick features (*n* = 10); (3) grey-level zone size matrix (GLZSM) (*n* = 11); (4) Gaussian transform (*n* = 303).

The ROI was placed by two experienced abdominal radiologists independently. Radiologist 1 (with 5 years of experience) performed the segmentation of all patients twice with a 6-month interval. Radiologist 2 (with 3 years of experience) performed the segmentation of all patients once. From the iodine-based MD images of venous phase, the two radiologists selected the slice with the largest axial diameter of CRC tumor and its adjacent upper and lower slices. Then, they manually outlined the boundary of the visible tumor on the selected slices via an open-source software ITK-SNAP (version 3.6.0) (17). The ROIs were required to include the area of necrosis and bleeding within the tumor and excluded perienteric fat and intestinal contents. To correct for acquisition-related differences of differing voxel resolutions in the two different CT scanners, voxel dimensions (mm) of each iodine-based MD image dataset were isotropically resampled to a common voxel spacing 0.5 × 0.5 × 0.5 mm^3^ (*x, y, z*) via linear interpolation algorithm (18, 19). Next, a total of 606 radiomics features for each CRC patient were extracted via Artificial Intelligent Kit (GE Healthcare) in concordance with the reference manual by the “Image Biomarker Standardization Initiative.” These features were divided into four groups: (1) first-order histogram features (*n* = 42); (2) second-order texture features: gray level co-occurrence matrix (GLCM) (*n* = 240), Haralick features (*n* = 10); (3) grey-level zone size matrix (GLZSM) (*n* = 11); and (4) Gaussian transform (*n* = 303). The inter-user variability for radiologist 1 and intra-user variability between radiologist 1 and radiologist 2 in tumor segmentation were analyzed via intraclass correlation coefficient (ICC) method [type: single rater; definition: absolute agreement; model: inter-user ICC: two-way random effects; intra-user ICC: two-way mixed effects (20)]. Details of radiomics features are described in Figure 2. The formulas of radiomics parameters are shown in Supplementary I.

### Feature Selection and Prediction Model Building

The dimensionality reduction of all features including clinical, CT-reported, and radiomics features was performed based on training dataset for further analysis. First, the radiomics features with both inter-user and intra-user stability (with ICC values >0.90) were selected via ICC analysis. Second, the continuous features with significant differences (*p* < 0.05) between MSI and MSS groups were selected by Student's *t*-test (for normally distributed data) or Mann–Whitney *U*-test (for non-normally distributed data). Then, the categorical features (gender, CEA, CA19-9, alcohol history, smoking history, hypertension history, diabetes history, family history of cancer, tumor location, CT-reported serous invasion, and CT-reported lymph node invasion) were encoded by using one-hot encoding. One-hot encoding uses N-bit state registers to encode N status, each of which has its own register bits, and at any time, only one of them is valid. One-hot encoding can convert the category variables into a form readily available to machine learning algorithms (21). For example, the “CT-reported tumor location” has three status, right colon, left colon, and rectum, which were coded as “1, 2, 3” in our study first. Then we used one-hot encoding method to encode right colon, left colon, and rectum as 100, 010, 001, respectively. One-hot encoding method can ensure that “1, 2, 3” represents the tumor location instead of the true value of 1, 2, or 3. Third, Spearman's rank correlation test was performed for each feature. Radiomics features with correlation coefficient ≥0.9 were selected into the following steps and then transferred with min–max standardization, whereas all features were normalized to a range of 0 to 1. Finally, the least absolute shrinkage and selection operator (LASSO) selection method was further used to identify the top-ranked and most valuable features to build the predictive model.

The selected features were applied to construct multiparameter logistic regression model to predict MSI status. The 5-fold cross-validation technique was used for model selection. The data in training set were divided into five subsets equally. Then, four subsets were selected each time to train, and the remaining one subset was used to test. By changing the subtest set in turn, five loss function values (*L*(*w*)) during the above five models would be obtained. The average value of *L*(*w*) was calculated. When the average *L*(*w*) reached a minimum value, the optimization of the logistic regression model would be completed, and the final model would be constructed. The details of *L*(*w*) are shown in Supplementary II. A nomogram based on clinical factors and radiomics score was generated via the proposed logistic regression model. The probability of MSI status defined as a nomogram score can be calculated for each patient by using the developed nomogram. The data from the Revolution CT equipment were used to establish and validate the radiomics model, and the data from the Discovery CT 750HD equipment were used to test the radiomics model.

### Radiomics Model Evaluation

**Figure 3:**
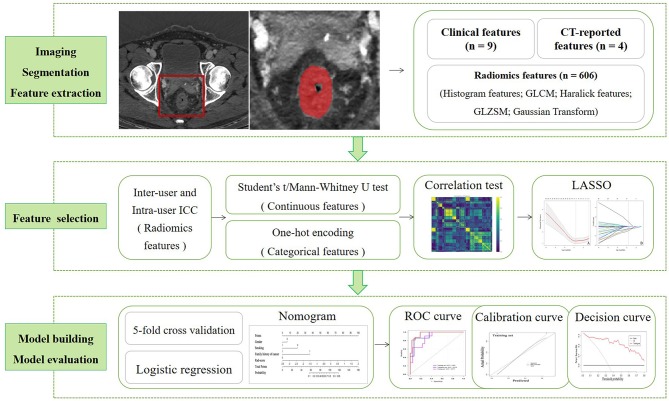
Workflow of radiomics analysis: iodine-based MD imaging and segmentation; feature extraction, feature selection, model building, and model evaluation. MD, material decomposition.

The discrimination performance was accessed by using receiver operating characteristic (ROC) curve analysis. The area under the ROC curve (AUC), accuracy, sensitivity, and specificity was calculated. DeLong's test was used to compare the statistically difference between AUCs. The calibration performance was tested by using the calibration curve accompanied by the Hosmer–Lemeshow test (H-L test). The calibration curves measure the consistency between the predicted MSI status probability and the actual MSI status probability. The H-L test assesses the goodness of fit of the prediction models. The clinical utility of radiomics model was assessed by using decision curve analysis. For decision curve, the horizontal axis indicates the threshold probability with a range of 0.0 to 1.0. The vertical axis indicates the clinical net benefit values. There are two reference lines defined under the assumption that all patients are diagnosed to be either MSI or MSS. A larger area under the decision curve suggests a better clinical utility. All statistical analyses were conducted with R software (version 3.6.0; https://www.r-project.org/). The workflow of radiomics analysis is shown in Figure 3.

## Results

### Inter-user and Intra-user Variabilities

The stable radiomics features (with ICC values >0.90) were as follows: 503 features between the two sets of measurements for radiologist 1, 568 features between the first measurement of radiologist 1 and radiologist 2, and 430 features between the second measurement of radiologist 1 and radiologist 2. Finally, 429 features were considered stable with both inter-user and intra-user stability. These 429 features obtained by radiologist 1 in the first measurement were used for the next analysis.

### Feature Selection and Radiomics Model Building

**Figure 4:**
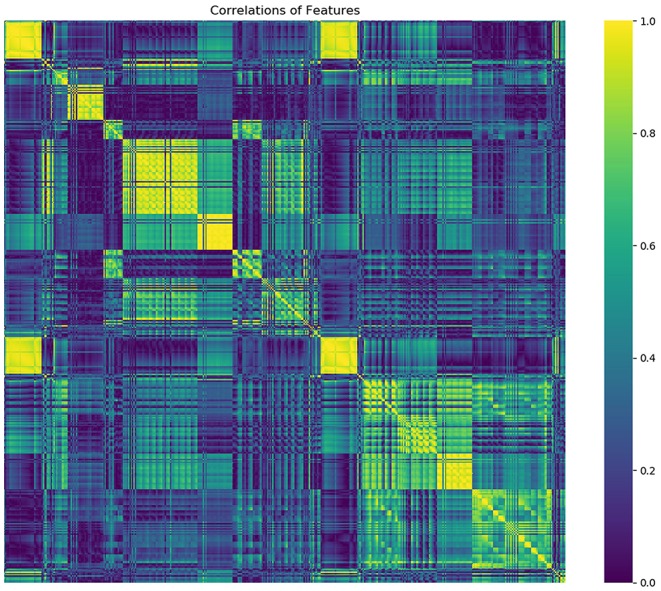
The heat map gives a good visual representation of the feature correlation. The *x* axis and *y* axis indicate features. Color scale on the right side displays the absolute value of the correlation coefficient (higher from 0 to 1, and from blue to yellow).

**Figure 5:**
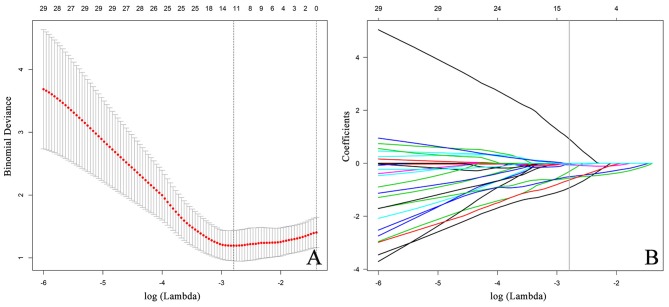
Feature selection using the LASSO algorithm. **(A)** The LASSO tuning parameter (lambda,λ) is iteratively updated by the optimization theory, and the optimal values of λ are indicated by the dotted vertical lines, a value λ of 0.0612 with log(λ) = −2.79 is chosen. **(B)** The LASSO algorithm performs the trend of the coefficients in the feature selection process. A coefficient profile plot is generated by violating the log (λ) sequence. The parameter λ is optimized by a five-fold cross-validation technique, and when the loss function reaches a minimum, 12 variables are selected. The 12 variables correspond to nine features including three clinical features and six radiomics features. The vertical line indicates the coefficient size of each variable and the corresponding log(λ) value when the model is optimal. LASSO, least absolute shrinkage and selection operator.

**Figure 6:**
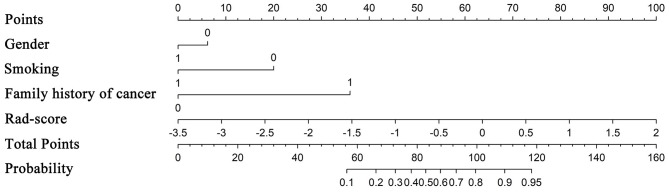
Nomogram based on clinical factors and radiomics score.

From a total of 429 radiomics features and 13 clinical or CT-reported features, the nine top-ranked features were finally selected for subsequent analysis: gender, smoking, family history of cancer, MaxIntensity, uniformity, GLCMEnergy_AllDirection_offset6_SD_Gaussian, GLCMEnergy_angle90_offset8_Gaussian, GLCMEntropy_AllDirection_offset8_Gaussian, and HaralickCorrelation_AllDirection_offset8_SD_Gaussian. The correlation heat map summarizes the correlations of features (Figure 4). Feature selection using the LASSO algorithm is shown in Figure 5. The nomogram based on clinical factors and radiomics score is shown in Figure 6.

$$\begin{array}{l} Rad-score=\\ -5.63e-01\times MaxIntensity\\ -5.08e-01\times uniformity\\ -1.76e-02\times GLCMEnergy\text{\_}AllDirection\text{\_}offset6\text{\_}SD\text{\_}Gaussian\\ -8.25e-02\times GLCMEnergy\text{\_}angle90\text{\_}offset8\text{\_}Gaussian\\ -3.70e-02\times GLCMEntropy\text{\_}AllDirection\text{\_}offset8\text{\_}Gaussian\\ -6.30e-01\times HaralickCorrelation\text{\_}AllDirection\text{\_}offset8\text{\_}SD\\ \text{\_}Gaussian \end{array}$$

### Radiomics Model Evaluation

**Figure 7:**
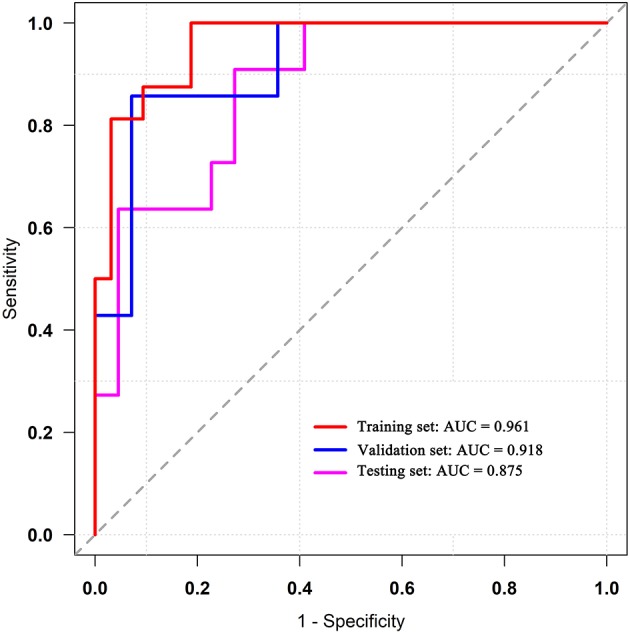
ROC curves of the radiomics models in training, validation, and testing sets. ROC, receiver operating characteristic.

**Figure 8:**
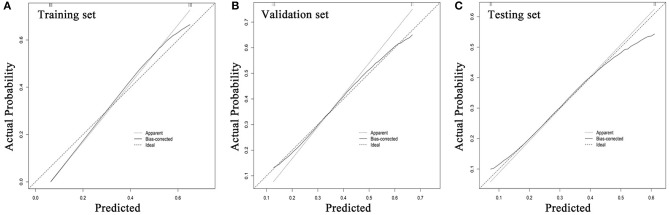
Calibration curves for radiomics models in training **(A)**, validation **(B)**, and testing sets **(C)**. The diagonal dashed reference line represents a perfect estimated MSI status by an ideal model. Solid lines represent estimated MSI status of the model. Good alignment of diagonal dashed reference line and solid line indicates a good performance. MSI, microsatellite instability.

**Figure 9:**
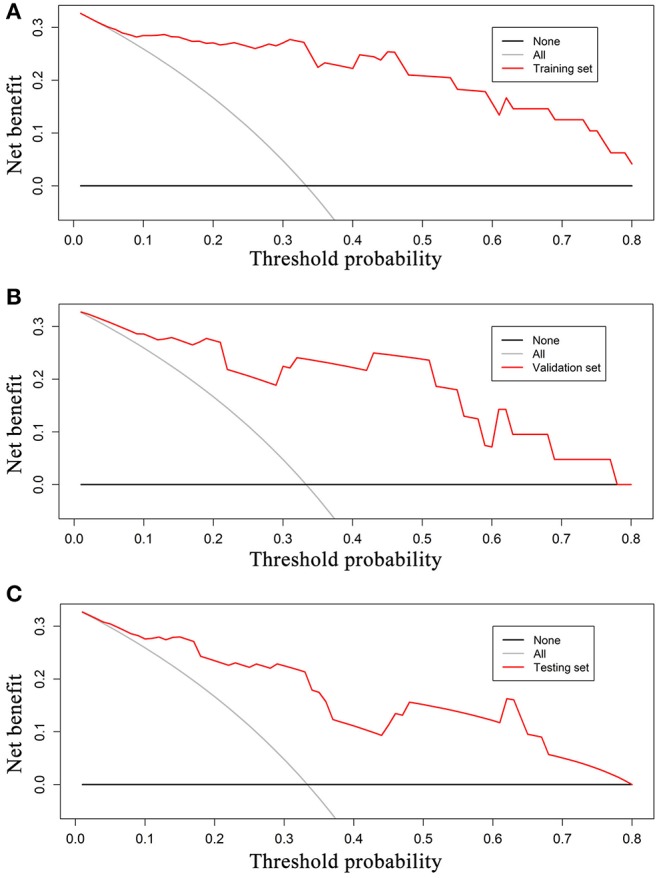
Decision curves for radiomics models in training **(A)**, validation **(B)**, and testing sets **(C)**. The grey reference line indicates the assumption that all patients are diagnosed to be MSI CRC; black dashed reference line indicates the assumption that all patients are diagnosed to be MSS CRC. In training, validation, testing sets, the net benefit is 17.44, 15.40, and 13.43, respectively. A larger area under the decision curve indicates a better clinical utility. MSI, microsatellite instability; CRC, colorectal cancer; MSS, microsatellite stability.

ROC analysis was applied to evaluate the model's performance for predicting MSI status. In the training set, AUC was 0.961 (95%CI [0.861, 0.996]; accuracy: 0.875; sensitivity: 1.000; specificity: 0.812); in the validation set, AUC was 0.918 (95%CI [0.714, 0.992]; accuracy: 0.875; sensitivity: 0.875; specificity: 0.857); and in the testing set, AUC was 0.875 (95%CI [0.715, 0.964]; accuracy: 0.788; sensitivity: 0.909; specificity: 0.727) (Table 2). DeLong's test revealed that above AUCs had no significant difference, which suggested that there was no overfitting among training, validation, and testing sets: *p* = 0.535 (Δ = 0.043, 95%CI [0.0416, 0.0894]) between the training and validation sets; *p* = 0.198 (Δ = 0.085, 95%CI [0.0266, 0.1476]) between the training and testing sets; and *p* = 0.631 (Δ = 0.042, 95%CI [0.0224, 0.1492]) between the validation and testing sets. The ROC curves are shown in Figure 7. Good calibrations of radiomics models for predicting MSI status in training, validation, and testing sets are shown in Figure 8. The H-L test was not significant (*p* > 0.05), demonstrating a good fit (training set: *p* = 0.462; validation set: *p* = 0.785; testing set: *p* = 0.568). The decision curves for radiomics models in training, validation, and testing sets (with net benefit of 17.44, 15.40, and 13.43, respectively) are presented in Figure 9.

**Table 2:** ROC analysis for predicting MSI status.

	**Revolution CT**		**Discovery CT 750HD**
	**Training set**	**Validation set**	**Testing set**
AUC	0.961	0.918	0.875
95%CI	[0.861, 0.996]	[0.714, 0.992]	[0.715, 0.964]
Accuracy	0.875	0.875	0.788
Sensitivity	1.000	0.875	0.909
Specificity	0.812	0.857	0.727

*ROC, receiver operating characteristic; AUC, area under the ROC curve; CI, confidence interval; MSI, microsatellite instability*.

## Discussion

In our study, we established a radiomics model based on iodine-based MD images to predict MSI status in CRC patients before surgery. We achieved a good diagnostic performance based on data from the Revolution CT equipment in both the training set (AUC, 0.961) and validation set (AUC, 0.918). And this radiomics model was also suitable for the iodine-based MD images acquired on another CT equipment (Discovery CT 750HD) although with slightly lower diagnostic performance (AUC, 0.875).

Iodine-based MD images can quantitatively reflect the vascularization of tissues. A clear relationship between blood supply and IC values calculated from iodine-based MD images has been confirmed; the richer blood supply is accompanied by a higher IC value (8, 22, 23). Our previous study has found that IC value of MSI CRC was significantly lower than that of MSS CRC and demonstrated the clinical value of using the IC values to distinguish MSI status with limited diagnostic performance (10). The measurement of IC in the previous study only reflected the average IC value in ROIs, and more information (such as tumor heterogeneity) was not evaluated. Therefore, radiomics approach, which can provide a wealth of complementary information of the images, should further extend our knowledge and improve the diagnosis (24–26). In our study, six radiomics features were finally selected as the most closely related features to the MSI status. For iodine-based MD images, the MaxIntensity generally represents the most abundant blood supply value within the predefined ROI. This may be explained by the biological characteristics of MSI CRC with less angiogenic phenotype confirmed by previous investigations (11, 27). The uniformity is a measure of the sum of the squares of each intensity value. From the perspective of image smoothness, the higher the intensity value, the higher the uniformity of the image. GLCMEnergy_AllDirection_offset6_SD_Gaussian and GLCMEnergy_angle90_offset8_Gaussian describe the uniformity of the intensity level distribution. GLCMEntropy_AllDirection_offset8_Gaussian describes the randomness of image values. It mainly calculates the average amount of information to encode image values. HaralickCorrelation_AllDirection_offset8_SD_Gaussian measures the linear dependency of grey levels of neighboring pixels; in other words, it measures the similarity of the grey levels in neighboring pixels and tells how correlated a pixel is to its neighbor over the whole image (28, 29). They have all served as recognized parameters to reflect tumor heterogeneity. We reviewed the biological differences between MSI and MSS tumors and tried to explain the imaging heterogeneity observed in this study. De Smedt et al. suggested that the morphological heterogeneity was the most striking feature to distinguish MSI from MSS CRC. Histologically, MSI CRC is often more inclined to present with a mixed morphological patterns including glandular, mucinous, and solid content, which caused the tumor heterogeneity (30). In addition, the higher incidence of internal heterogeneity in MSI CRC may also be explained by a higher density of tumor-infiltrating lymphocytes and a lower cell proliferation rate than MSS CRC (31, 32). Our results that the imaging heterogeneity was a biomarker for MSI tumors were consistent with those of previous studies (14). During radiomics analysis, integrating diverse clinical features plays an important role in improving the performance of the diagnostic model. We recorded the clinical features and CT reported features, which were discrete data except for age and tumor size. We used the one-hot encoding to process category variables, with the main benefits of one-hot encoding as follows: (1) to solve the problem that the classifier is not good at processing category data; (2) to a certain extent also play a role in expanding features; and (3) to choose the most representative new features. In our study, we found that the gender, smoking, and family history of cancer were closely related with the MSI status in CRC patients, and further explorations were required based on larger samples (13, 33).

Radiomics analysis is a promising method to unveil large amount of tumor features hidden in medical images. However, previous studies have reported that the repeatability of radiomics features can be influenced by different CT scanners (34). Our study included data from two different DECT scanners including Revolution CT and Discovery CT 750HD. We first used the data obtained from the Revolution CT scanner to establish a model for preoperatively predicting MSI status; the AUCs of training and validation sets were 0.961 and 0.918, respectively. Subsequently, we analyzed whether this radiomics model was suitable for another DECT scanner (Discovery CT 750HD), and we found that the performance was good with AUC of 0.875. Our results suggested that the radiomics model established in this study was applicable to both Revolution CT and Discovery CT 750HD, and this might be attributed to the stability of the iodine quantification, and that there is little effect of various DECT scanners and acquisition parameters on iodine density (35). Hence, further studies are recommended to focus on the radiomics analysis of iodine-based MD images with DECT imaging.

Our study has several limitations. First, the study was retrospective and may result in inherent biases. Second, although IHC test is a reliable way to assess MSI status, the PCR should still be recommended. Third, only a handful of patients were analyzed owing to the low incidence rate of MSI in CRC patients. Further studies are required using a larger sample. Fourth, only three slices of CT images were analyzed, and we plan to compare the performance of using three slices and whole tumors in future investigations. Fifth, some discrepancies caused by manually outlined ROIs are unavoidable, even though we had made efforts to minimize the bias by using two trained radiologists. Sixth, our data were only from a single center. In the future, we will try to collect multicenter data to reinforce the conclusions of our study.

In conclusion, radiomics analysis based on iodine-based MD images with DECT imaging can provide a relatively high diagnostic value for predicting MSI status in CRC patients. This study provides insight into the potential applications of using radiomics analysis of iodine-based MD images produced via DECT in predicting MSI status, and its usefulness for preoperatively providing more information in CRC clinical outcome and treatment decision making.

## Data Availability Statement

The datasets generated for this study are available on request to the corresponding author.

## Ethics Statement

The studies involving human participants were reviewed and approved by The First Affiliated Hospital of Dalian Medical University. Written informed consent for participation was not required for this study in accordance with the national legislation and the institutional requirements.

## Author Contributions

Data analysis and interpretation, study design, manuscript writing, and manuscript approval were performed by JW, QZ, and AL, and they are accountable for all aspects of the work. CT data and pathological data analysis and interpretation, statistical analysis, and manuscript approval were performed by YZ. CT data analysis and manuscript approval were performed by YL and AC. Statistical analysis and manuscript approval were performed by XL, TW, JL, and YG.

### Conflict of Interest

The authors declare that the research was conducted in the absence of any commercial or financial relationships that could be construed as a potential conflict of interest.

## Abbreviations

AUC: area under the ROC curve
CRC: colorectal cancer
DECT: dual-energy computed tomography
DICOM: digital imaging data and communications in medicine
GLCM: gray level co-occurrence matrix
GLZSM: grey-level zone size matrix
IC: iodine concentration
IHC: immunohistochemistry
LASSO: least absolute shrinkage and selection operator
MD: material decomposition
MMR: mismatch repair
MSI: microsatellite instability
MSS: microsatellite stability
PCR: polymerase chain reaction
ROC: receiver operating characteristic
ROI: region of interest.
