# Supplementary material for: Radiomics Analysis of Iodine-Based Material Decomposition Images With Dual-Energy Computed Tomography Imaging for Preoperatively Predicting Microsatellite Instability Status in Colorectal Cancer
Source: Front Oncol. 2019 Nov 22;9:1250. doi: 10.3389/fonc.2019.01250 (PMC6883423; doi:10.3389/fonc.2019.01250)
Supplement: Supplementary file 1 [file Table_1.DOCX]

**Supplementary I：Radiomics Parameters Description**

[1. Histogram Features 1](#_Toc476144786)

[1.1 MinIntensity 1](#_Toc476144787)

[1.2 MaxIntensity 1](#_Toc476144788)

[1.3 MedianIntensity 1](#_Toc476144789)

[1.4 MeanValue 1](#_Toc476144790)

[1.5](#_Toc476144791) [StandardDeviation 1](#_Toc476144791)

[1.6 MeanDeviation 2](#_Toc476144792)

[1.7 RelativeDeviation 2](#_Toc476144793)

[1.8 Variance 2](#_Toc476144794)

[1.9 Range 2](#_Toc476144795)

[1.10 VolumeCount 2](#_Toc476144796)

[1.11 VoxelValueSum 2](#_Toc476144797)

[1.12](#_Toc476144798) [RootMeanSquare (RMS) 2](#_Toc476144798)

[1.13 Skewness 3](#_Toc476144799)

[1.14 Kurtosis 3](#_Toc476144800)

[1.15 Uniformity 3](#_Toc476144801)

[1.16 Histogram Energy 4](#_Toc476144802)

[1.17 Histogram Entropy 4](#_Toc476144803)

[1.18 FrequencySize 4](#_Toc476144804)

[1.19 Percentile 4](#_Toc476144805)

[1.20 Quantile 5](#_Toc476144806)

[2. Texture Features 5](#_Toc476144807)

[2.1](#_Toc476144824) [Gray Level Co-occurrence Matrix (GLCM) 6](#_Toc476144824)

[2.1.1 GLCMEnergy 6](#_Toc476144825)

[2.1.2 GLCMEntropy 7](#_Toc476144826)

[2.1.3 GLCMInertia 8](#_Toc476144827)

[2.1.4 Correlation 9](#_Toc476144828)

[2.1.5 InverseDifferenceMoment 1](#_Toc476144829)0

[2.1.6 ClusterShade 12](#_Toc476144830)

[2.1.7 ClusterProminence 13](#_Toc476144830)

[2.1.8 HaralickCorrelation 1](#_Toc476144831)5

2.2 Haralick features 17

[2.2.1 HaraEntroy 18](#_Toc476144832)

[2.2.2 AngularSecondMoment 18](#_Toc476144833)

[2.2.3 Contrast 18](#_Toc476144834)

[2.2.4 HaraVariance 1](#_Toc476144835)9

[2.2.5 SumAverage 1](#_Toc476144836)9

[2.2.6 SumVariance 1](#_Toc476144837)9

[2.2.7 SumEntropy 1](#_Toc476144838)9

[2.2.8 DifferenceVariance 1](#_Toc476144839)9

[2.2.9 DifferenceEntropy 20](#_Toc476144840)

[2.2.10 InverseDifferenceMoment 20](#_Toc476144841)

[3. Grey Level Zone Size Matrix (GLZSM) 20](#_Toc476144842)

[3.1 SmallAreaEmphasis 21](#_Toc476144843)

[3.2 LargeAreaEmphasis 21](#_Toc476144844)

[3.3 IntensityVariability 21](#_Toc476144845)

[3.4 SizeZoneVariability 22](#_Toc476144846)

[3.5 ZonePercentage 22](#_Toc476144847)

[3.6 LowIntensityEmphasis 22](#_Toc476144848)

[3.7 HighIntensityEmphasis 22](#_Toc476144849)

[3.8 LowIntensitySmallAreaEmphasis 22](#_Toc476144850)

[3.9 HighIntensitySmallAreaEmphasis 22](#_Toc476144851)

[3.10 LowIntensityLargeAreaEmphasis](#_Toc476144851) 22

[3.11 HighIntensityLargeAreaEmphasis 23](#_Toc476144851)

[4. Gaussian Transform 2](#_Toc476144842)[3](#_Toc476144851)

# **Histogram Features**

Histogram parameters are concerned with properties of individual pixels. They describe the distribution of voxel intensities within the CT image through commonly used and basic metrics. Let $X$ denote the three dimensional image matrix with $N$ voxels, and let $P$ denote the first order histogram divided by $N_{l}$ discrete intensity levels. The following first order statistics were extracted:

## **1.1 MinIntensity:**

The minimum intensity value of $X$.

## **1.2 MaxIntensity:**

The maximum intensity value of $X$.

## **1.3 MedianIntensity:**

The median intensity value of $X$.

## **1.4 MeanValue:**

The mean measures the average value of the intensity values.

$$mean=\frac{1}{N}\sum_{i}^{N} X(i)$$

## **1.5 StandardDeviation:**

Is a measure that is used to quantify the amount of variation or dispersion of a set of data values.

$$standard deviation=\left( \frac{1}{N-1}\sum_{i=1}^{N} \left( X\left( i \right)-\bar{X} \right)^{2} \right)^{1/2}$$

Where $\bar{X}$ is the mean of $X$.

## **1.6 MeanDeviation:**

The mean of the absolute deviations of all voxel intensities around the mean intensity value.

## **1.7 RelativeDeviation:**

Let $\overline{X}$ denote the mean of a set of quantities $X_{i}$, then the relative deviation is defined by:

$$\frac{{\Delta X}_{i}}{\overline{X}}= \frac{\left| X_{i}-\overline{X} \right|}{\overline{X}}$$

## **1.8 Variance:**

Is the average of the squared differences from the Mean.

$$variance=\frac{1}{N-1}\sum_{i=1}^{N} {(X\left( i \right)-\bar{X})}^{2}$$

Where $\overline{X}$ is the mean of $X$.

## **1.9 Range:**

The range of intensity values of $X$.

## **1.10 VolumeCount:**

Describe the size of the ROI.

## **1.11 VoxelValueSum:**

Represents the Sum calculations for voxels in the ROI.

## **1.12 RootMeanSquare (RMS):**

$$RMS=\sqrt{\frac{\sum_{i}^{N} {X(i)}^{2}}{N}}$$

## **1.13 Skewness:**

Represents the degree of asymmetric distribution in the image histogram, this means that in some distribution of data, the right and the left of the distribution are perfect mirror images of one another, the mean, median and mode are all measures of the center of a set of data. The Skewness of the data can be determined by how these quantities are related to one another.

$$skewness=\frac{\frac{1}{N}\sum_{i=1}^{N} \left( X\left( i \right)-\bar{X} \right)^{3}}{\left( \sqrt{\frac{1}{N}\sum_{i=1}^{N} \left( X\left( i \right)-\bar{X} \right)^{2}} \right)^{3}}$$

Where $\bar{X}$ is the mean of $X$.

## **1.14 Kurtosis:**

Kurtosis is a measure of whether the data are heavy-tailed or light-tailed relative to a normal distribution. That is, data sets with high kurtosis tend to have heavy tails, or outliers. Data sets with low kurtosis tend to have light tails, or lack of outliers. A uniform distribution would be the extreme case.

$$kurtosis=\frac{\frac{1}{N}\sum_{i=1}^{N} \left( X\left( i \right)-\bar{X} \right)^{4}}{\left( \sqrt{\frac{1}{N}\sum_{i=1}^{N} \left( X\left( i \right)-\bar{X} \right)^{2}} \right)^{2}}$$

Where $\overline{X}$ is the mean of $X$.

## **1.15 Uniformity:**

$$uniformity=\sum_{i=1}^{N_{l}} {P\left( i \right)}^{2}$$

## **1.16 Histogram Energy:**

The energy feature measures the uniformity of the intensity level distribution. If the value is high, then the distribution is to a small number of intensity levels. Energy can be defined as:

$$energy = \sum_{i}^{N} {X\left( i \right)}^{2}$$

## **1.17 Histogram Entropy:**

The entropy measures the randomness of the distribution of the coefficients values over the intensity levels. If the value of entropy is high, then the distribution is among more intensity levels in the image. This measurement is the inverse of energy. A simple image has low entropy while a complex image has high entropy. Entropy can be defined as:

$$entropy=-\sum_{i=1}^{N_{l}} P\left( i \right){log}_{2} P\left( i \right)$$

## **1.18 FrequencySize:**

The ratio of the frequency of each object to the total frequency.

## **1.19 Percentile:**

The percentile (p%) is defined as that value of the brightness $a$:

$$P\left( a \right)=p\%$$

or equivalently:

$$\int_{-\infty}^{a} P\left( a \right)=p\%$$

The P-th percentile of a list of N ordered values (sorted from least to greatest) is the smallest value in the list such that P percent of the data is less than or equal to that value. This is obtained by first calculating the ordinal rank and then taking the value from the ordered list that corresponds to that rank. The ordinal rank n is calculated using this formula:

$$n=\frac{P}{100}*$$

The 19 Percentiles are extracted:

Percentile5, Percentile10, Percentile15, Percentile20, Percentile25, Percentile30,

Percentile35, Percentile40, Percentile45, Percentile50, Percentile55, Percentile60,

Percentile65, Percentile70, Percentile75, Percentile80, Percentile85, Percentile90,

Percentile95

## **1.20 Quantile:**

For a finite population of *N* equally probable values indexed 1, …, *N* from lowest to highest, the k-th q-quantile of this population can equivalently be computed via the value of:

$$I_{p}=\frac{N*k}{q}$$

The 5 Quantiles are extracted:

Quantile0.025, Quantile0.25, Quantile0.5, Quantile0.75, Quantile0.975.

# **Texture Features**

Texture is one of the important characteristics used in identifying objects or regions of interest in an image, texture represents the appearance of the surface and how its elements are distributed.

## **2.1 Gray Level Co-occurrence Matrix (GLCM):**

The Grey level co-occurrence matrix (GLCM) $\boldsymbol{P (i, j|\theta, d)}$represents the joint probability of certain sets of pixels having certain grey-level values. It calculates how many times a pixel with grey-level i occurs jointly with another pixel having a grey value j. By varying the displacement vector d between each pair of pixels.

The rotation angle of an offset: 0°, 45°, 90°, 135° and displacement vectors (distance to the neighbor pixel: 1, 2, 3 ...), different co-occurrence distributions from the same image of reference. GLCM of an image is computed using displacement vector d defined by its radius, (distance or count to the next adjacent neighbor preferably is equal to one) and rotational angles.


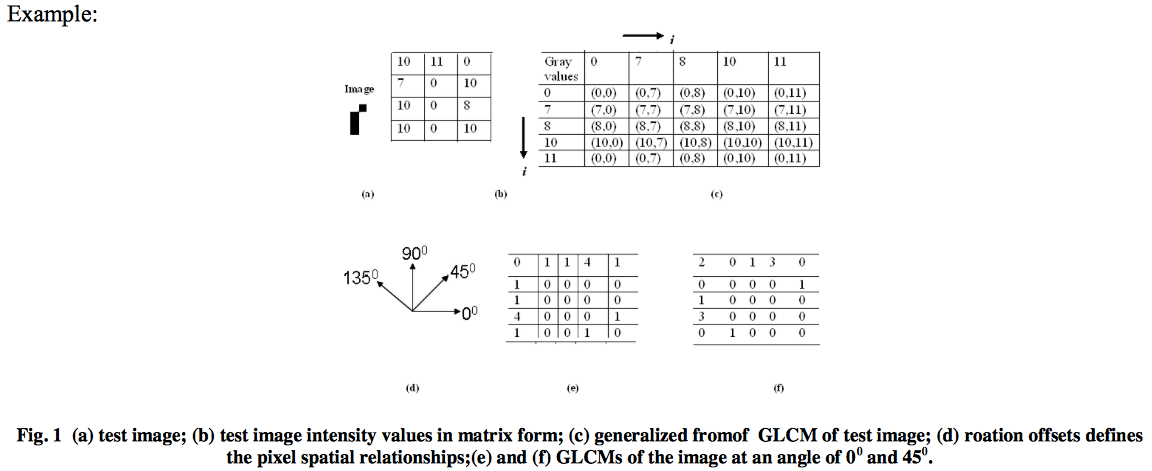


### 2.1.1 GLCMEnergy

Formula:

$$\sum_{i,j} {g\left( i,j \right)}^{2}$$

g is a GLCM

Where i,j are the spatial coordinates of g (i,j).

*Angle=All Direction, 0, 45, 90, 135, All_SD;

*Offset=1, 2, 4, 6, 8

The 30 features of GLCMEnergy are extracted:

GLCMEnergy_AllDirection_offset1, GLCMEnergy_AllDirection_offset1_SD, GLCMEnergy_angle0_offset1, GLCMEnergy_angle45_offset1, GLCMEnergy_angle90_offset1, GLCMEnergy_angle135_offset1,

GLCMEnergy_AllDirection_offset2, GLCMEnergy_AllDirection_offset2_SD, GLCMEnergy_angle0_offset2, GLCMEnergy_angle45_offset2, GLCMEnergy_angle90_offset2, GLCMEnergy_angle135_offset2,

GLCMEnergy_AllDirection_offset4, GLCMEnergy_angle0_offset4, GLCMEnergy_angle45_offset4, GLCMEnergy_angle90_offset4, GLCMEnergy_angle135_offset4, GLCMEnergy_AllDirection_offset4_SD,

GLCMEnergy_AllDirection_offset6, GLCMEnergy_AllDirection_offset6_SD, GLCMEnergy_angle0_offset6, GLCMEnergy_angle45_offset6, GLCMEnergy_angle90_offset6, GLCMEnergy_angle135_offset6,

GLCMEnergy_AllDirection_offset8, GLCMEnergy_angle0_offset8, GLCMEnergy_angle45_offset8, GLCMEnergy_angle90_offset8, GLCMEnergy_angle135_offset8, GLCMEnergy_AllDirection_offset8_SD

### 2.1.2 GLCMEntropy

Formula:

$$-\sum_{i,j} g\left( i,j \right){log}_{2} \left( i,j \right)$$

*Angle=All Direction, 0, 45, 90, 135, All_SD;

*Offset=1, 2, 4, 6, 8

The 30 features of GLCMEntropy are extracted:

GLCMEntropy_AllDirection_offset1, GLCMEntropy_AllDirection_offset1_SD, GLCMEntropy_angle0_offset1, GLCMEntropy_angle45_offset1, GLCMEntropy_angle90_offset1, GLCMEntropy_angle135_offset1,

GLCMEntropy_AllDirection_offset2, GLCMEntropy_AllDirection_offset2_SD, GLCMEntropy_angle0_offset2, GLCMEntropy_angle45_offset2, GLCMEntropy_angle90_offset2, GLCMEntropy_angle135_offset2,

GLCMEntropy_AllDirection_offset4, GLCMEntropy_AllDirection_offset4_SD, GLCMEntropy_angle0_offset4, GLCMEntropy_angle45_offset4, GLCMEntropy_angle90_offset4, GLCMEntropy_angle135_offset4,

GLCMEntropy_AllDirection_offset6,GLCMEntropy_AllDirection_offset6_SD, GLCMEntropy_angle0_offset6, GLCMEntropy_angle45_offset6, GLCMEntropy_angle90_offset6, GLCMEntropy_angle135_offset6, GLCMEntropy_AllDirection_offset8, GLCMEntropy_AllDirection_offset8_SD, GLCMEntropy_angle0_offset8, GLCMEntropy_angle45_offset8, GLCMEntropy_angle90_offset8, GLCMEntropy_angle135_offset8

### 2.1.3 GLCMInertia

Formula:

$$\sum_{i,j} \left( \left( i,j \right)^{2}g \left( i,j \right) \right)$$

*Angle=All Direction, 0, 45, 90, 135, All_SD;

*Offset=1, 2, 4, 6, 8

The 30 features of GLCMInertia are extracted:

Inertia_AllDirection_offset1, Inertia _AllDirection_offset1_SD,

Inertia _angle0_offset1, Inertia _angle45_offset1,

Inertia _angle90_offset1, Inertia _angle135_offset1,

Inertia_AllDirection_offset2, Inertia _AllDirection_offset2_SD,

Inertia _angle0_offset2, Inertia _angle45_offset2,

Inertia _angle90_offset2, Inertia _angle135_offset2,

Inertia _AllDirection_offset4, Inertia _AllDirection_offset4_SD,

Inertia _angle0_offset4, Inertia _angle45_offset4,

Inertia _angle90_offset4, Inertia _angle135_offset4,

Inertia_AllDirection_offset6, Inertia _AllDirection_offset6_SD,

Inertia _angle0_offset6, Inertia _angle45_offset6,

Inertia _angle90_offset6, Inertia _angle135_offset6,

Inertia _AllDirection_offset8, Inertia _AllDirection_offset8_SD,

Inertia _angle0_offset8, Inertia _angle45_offset8,

Inertia _angle90_offset8, Inertia _angle135_offset8

### 2.1.4 Correlation

Formula:

$$-\sum_{i, j} \frac{\left( i-\mu\right)\left( j-\mu\right)g\left( i, j \right)}{\sigma^{2}}$$

*Angle=All Direction, 0, 45, 90, 135, All_SD;

*Offset=1, 2, 4, 6, 8

The 30 features of Correlation are extracted:

Correlation_AllDirection_offset1, Correlation _AllDirection_offset1_SD,

Correlation _angle0_offset1, Correlation _angle45_offset1,

Correlation _angle90_offset1, Correlation _angle135_offset1,

Correlation_AllDirection_offset2, Correlation _AllDirection_offset2_SD,

Correlation _angle0_offset2, Correlation _angle45_offset2,

Correlation _angle90_offset2, Correlation _angle135_offset2,

Correlation _AllDirection_offset4, Correlation _AllDirection_offset4_SD,

Correlation _angle0_offset4, Correlation _angle45_offset4,

Correlation _angle90_offset4, Correlation _angle135_offset4,

Correlation_AllDirection_offset6, Correlation _AllDirection_offset6_SD,

Correlation _angle0_offset6, Correlation _angle45_offset6,

Correlation _angle90_offset6, Correlation _angle135_offset6,

Correlation _AllDirection_offset8, Correlation _AllDirection_offset8_SD,

Correlation _angle0_offset8, Correlation _angle45_offset8,

Correlation _angle90_offset8, Correlation _angle135_offset8

### 2.1.5 InverseDifferenceMoment

Formula:

$$\mu\sum\sum\frac{1}{1+\left( i-j \right)^{2}}g\left( i, j \right)$$

*Angle=All Direction, 0, 45, 90, 135, All_SD;

*Offset=1, 2, 4, 6, 8

The 30 features of InverseDifferenceMoment are extracted:

InverseDifferenceMoment_AllDirection_offset1, InverseDifferenceMoment_AllDirection_offset1_SD,

InverseDifferenceMoment_angle0_offset1,

InverseDifferenceMoment_angle45_offset1,

InverseDifferenceMoment_angle90_offset1,

InverseDifferenceMoment_angle135_offset1,

InverseDifferenceMoment_AllDirection_offset2, InverseDifferenceMoment_AllDirection_offset2_SD,

InverseDifferenceMoment_angle0_offset2,

InverseDifferenceMoment_angle45_offset2,

InverseDifferenceMoment_angle90_offset2,

InverseDifferenceMoment_angle135_offset2,

InverseDifferenceMoment_AllDirection_offset4,

InverseDifferenceMoment_AllDirection_offset4_SD,

InverseDifferenceMoment_angle0_offset4,

InverseDifferenceMoment_angle45_offset4,

InverseDifferenceMoment_angle90_offset4,

InverseDifferenceMoment_angle135_offset4,

InverseDifferenceMoment_AllDirection_offset6, InverseDifferenceMoment_AllDirection_offset6_SD,

InverseDifferenceMoment_angle0_offset6,

InverseDifferenceMoment_angle45_offset6,

InverseDifferenceMoment_angle90_offset6,

InverseDifferenceMoment_angle135_offset6,

InverseDifferenceMoment_AllDirection_offset8,

InverseDifferenceMoment_AllDirection_offset8_SD,

InverseDifferenceMoment_angle0_offset8,

InverseDifferenceMoment_angle45_offset8,

InverseDifferenceMoment_angle90_offset8,

InverseDifferenceMoment_angle135_offset8

### 2.1.6 ClusterShade

Formula:

$$\sum_{i, j} \left( \left( i-\mu\right)+\left( j-\mu\right) \right)^{3}g\left( i, j \right)$$

*Angle=All Direction, 0, 45, 90, 135, All_SD;

*Offset=1, 2, 4, 6, 8

The 30 features of ClusterShade are extracted:

ClusterShade_AllDirection_offset1, ClusterShade_AllDirection_offset1_SD,

ClusterShade_angle0_offset1, ClusterShade_angle45_offset1,

ClusterShade_angle90_offset1, ClusterShade_angle135_offset1,

ClusterShade_AllDirection_offset2, ClusterShade_AllDirection_offset2_SD,

ClusterShade_angle0_offset2, ClusterShade_angle45_offset2,

ClusterShade_angle90_offset2, ClusterShade_angle135_offset2,

ClusterShade_AllDirection_offset4, ClusterShade_AllDirection_offset4_SD,

ClusterShade_angle0_offset4, ClusterShade_angle45_offset4,

ClusterShade_angle90_offset4, ClusterShade_angle135_offset4,

ClusterShade_AllDirection_offset6, ClusterShade_AllDirection_offset6_SD,

ClusterShade_angle0_offset6, ClusterShade_angle45_offset6,

ClusterShade_angle90_offset6, ClusterShade_angle135_offset6,

ClusterShade_AllDirection_offset8, ClusterShade_AllDirection_offset8_SD,

ClusterShade_angle0_offset8, ClusterShade_angle45_offset8,

ClusterShade_angle90_offset8, ClusterShade_angle135_offset8

### 2.1.7 ClusterProminence

Formula:

$$\sum_{i, j} \left( \left( i-\mu\right)+\left( j-\mu\right) \right)^{4}g\left( i, j \right)$$

*Angle=All Direction, 0, 45, 90, 135, All_SD;

*Offset=1, 2, 4, 6, 8

The 30 features of ClusterProminence are extracted:

ClusterProminence_AllDirection_offset1, ClusterProminence_AllDirection_offset1_SD,

ClusterProminence_angle0_offset1,

ClusterProminence_angle45_offset1,

ClusterProminence_angle90_offset1,

ClusterProminence_angle135_offset1,

ClusterProminence_AllDirection_offset2, ClusterProminence_AllDirection_offset2_SD,

ClusterProminence_angle0_offset2,

ClusterProminence_angle45_offset2,

ClusterProminence_angle90_offset2,

ClusterProminence_angle135_offset2,

ClusterProminence_AllDirection_offset4,

ClusterProminence_AllDirection_offset4_SD,

ClusterProminence_angle0_offset4,

ClusterProminence_angle45_offset4,

ClusterProminence_angle90_offset4,

ClusterProminence_angle135_offset4,

ClusterProminence_AllDirection_offset6, ClusterProminence_AllDirection_offset6_SD,

ClusterProminence_angle0_offset6,

ClusterProminence_angle45_offset6,

ClusterProminence_angle90_offset6,

ClusterProminence_angle135_offset6,

ClusterProminence_AllDirection_offset8,

ClusterProminence_AllDirection_offset8_SD,

ClusterProminence_angle0_offset8,

ClusterProminence_angle45_offset8,

ClusterProminence_angle90_offset8,

ClusterProminence_angle135_offset8

### 2.1.8 HaralickCorrelation

Formula:

$$-\sum_{i, j} \frac{\left( i, j \right)g\left( i, j \right)-\mu_{t}^{2}}{\sigma_{t}^{2}}$$

where $\mu_{t}$ and $\sigma_{t}$ are the mean and standard deviation of the row (or column, due to

symmetry) sums.

*Angle=All Direction, 0, 45, 90, 135, All_SD;

*Offset=1, 2, 4, 6, 8

The 30 features of HaralickCorrelation are extracted:

HaralickCorrelation_AllDirection_offset1, HaralickCorrelation_AllDirection_offset1_SD,

HaralickCorrelation_angle0_offset1,

HaralickCorrelation_angle45_offset1,

HaralickCorrelation_angle90_offset1,

HaralickCorrelation_angle135_offset1,

HaralickCorrelation_AllDirection_offset2, HaralickCorrelation_AllDirection_offset2_SD,

HaralickCorrelation_angle0_offset2,

HaralickCorrelation_angle45_offset2,

HaralickCorrelation_angle90_offset2,

HaralickCorrelation_angle135_offset2,

HaralickCorrelation_AllDirection_offset4,

HaralickCorrelation_AllDirection_offset4_SD,

HaralickCorrelation_angle0_offset4,

HaralickCorrelation_angle45_offset4,

HaralickCorrelation_angle90_offset4,

HaralickCorrelation_angle135_offset4,

HaralickCorrelation_AllDirection_offset6, HaralickCorrelation_AllDirection_offset6_SD,

HaralickCorrelation_angle0_offset6,

HaralickCorrelation_angle45_offset6,

HaralickCorrelation_angle90_offset6,

HaralickCorrelation_angle135_offset6,

HaralickCorrelation_AllDirection_offset8,

HaralickCorrelation_AllDirection_offset8_SD,

HaralickCorrelation_angle0_offset8,

HaralickCorrelation_angle45_offset8,

HaralickCorrelation_angle90_offset8,

HaralickCorrelation_angle135_offset8

## **2.2 Haralick features**

| **Formula** | **Description** |
| --- | --- |
| $P_{ij}=P(i,j)$ | Matrix of relative frequencies with which two neighborinig resolution cells separated by distance d occur on the image |
| $p_{ij}=\frac{P(i,j)}{R}=(i,j)$ | The entry in the normalized gray-tone spatial dependence matrix. |
| $N_{g}$ | The number of distinct gray levels in the quantized image (the EBImage haralick.nbins parameter in the function computerFeatures.haralick defaults to 32 gray levels) |
| $R$ | Normalization constant, the number of neighboring resolution cell pairs used in computing a particular gray-tone spatial-dependence matrix. |
| $R=\sum_{i=1}^{N_{g}} \sum_{j=1}^{N_{g}} P(i,j)$ | Sum of all elements of co-occurrence frequency matrix |
| $p(i,j)=\frac{P(i,j)}{R}$ | Co-occurrence probability matrix. |
| $p_{x}(i)=\sum_{j=1}^{N_{g}} p(i,j)$ | $i$-th entry in the marginal-probability matrix obtained by summing the rows of $p(i,j)$. |
| $p_{y}(j)=\sum_{i=1}^{N_{g}} p(i,j)$ | $j$-th entry in the marginal-probability matrix obtained by summing the columns of $p(i,j)$. |
| $p_{x+y}(k)=\sum_{i=1}^{N_{g}} \sum_{j=1}^{N_{g}} \delta_{i+j,k}p(i,j)$，$k=2,3,...,2N_{g}$ |  |
| $p_{x-y}(k)=\sum_{i=1}^{N_{g}} \sum_{j=1}^{N_{g}} \delta_{\vert i-j\vert,k}p(i,j)$，$k=0,1,...,N_{g}-1$ |  |

### 2.2.1 HaraEntropy

Formula:

$$f=- \sum_{i=1}^{N_{g}} \sum_{j=1}^{N_{g}} p\left( i, j \right)\log\left( p\left( i, j \right) \right)$$

### 2.2.2 Angular Second Moment

Formula:

$$f= \sum_{i=1}^{N_{g}} \sum_{j=1}^{N_{g}} \left( \frac{P\left( i, j \right)}{R} \right)^{2} = \sum_{i} \sum_{j} p\left( i, j \right)^{2}$$

### 2.2.3 Contrast

The contrast feature, is a difference moment of the P matrix and is a measure of the contrast or the amount of local variations present in the image.

Formula:

$$f=\sum_{k=0}^{N_{g}-1} k^{2}\left\{ \sum_{i=1}^{N_{g}} \sum_{j=1}^{N_{g}} \delta_{\left| i-j \right|,k}p\left( i, j \right) \right\} = \sum_{k=0}^{N_{g}-1} k^{2}p_{x-y}\left( k \right)$$

### 2.2.4 Haralick Variance

Formula:

$$f=\sum_{i=1}^{N_{g}} \sum_{j=1}^{N_{g}} \left( i-\mu\right)^{2}p\left( i, j \right)$$

### 2.2.5 SumAverage

Formula:

$$f=\sum_{i=2}^{{2N}_{g}} ip_{x+y}\left( i \right)$$

### 2.2.6 SumVariance

Formula:

$$f=\sum_{i=2}^{{2N}_{g}} \left( i-f_{SumEntropy} \right)^{2}p_{x+y}\left( i \right)$$

### 2.2.7 SumEntropy

Formula:

$$f=-\sum_{i=2}^{{2N}_{g}} p_{x+y}\left( i \right)log\left( p_{x+y}\left( i \right) \right)$$

### 2.2.8 Difference Variance

Formula:

$$f=variance of p_{x-y}$$

### 2.2.9 Difference Entropy

Formula:

$$f=-\sum_{i=0}^{N_{g}-1} p_{x-y}\left( i \right)log\left( p_{x-y}\left( i \right) \right)$$

### 2.2.10 Inverse Difference Moment

Formula:

$$f=\sum_{i=1}^{N_{g}} \sum_{j=1}^{N_{g}} \frac{1}{1+\left( i-j \right)^{2}}p\left( i, j \right)$$

# **Grey Level Zone Size Matrix (GLZSM)**

The gray level Size Zone Matrix (SZM) is the starting point of Thibault matrices. For a texture image f with N gray levels, it is denoted GSf(s, g) and provides a statistical representation by the estimation of a bivariate conditional probability density function of the image distribution values. It is calculated according to the pioneering Run Length Matrix principle: the value of the matrix GSf(s, g) is equal to the number of zones of size s and of gray level g. The resulting matrix has a fixed number of lines equal to N, the number of gray levels, and a dynamic number of columns, determined by the size of the largest zone as well as the size quantization.

The more homogeneous the texture, the wider and flatter the matrix. SZM does not required computation in several directions. However, it has been empirically proved that the degree of gray level quantization still has an important impact on the texture classification performance.

Let P define the GLSZM of a quantized volume V (x,y,z) with isotropic voxel size. *P(i,j)* represents the number of 3D zones of gray-levels *i* and of size j in V , *Ng* represents the pre-defined number of quantized gray-levels set in V, and *Lz* represents the size of the largest zone (of any gray-level) in V. One GLSZM of size *Ng*× *Lz* is computed per volume V by adding up all possible largest zone-sizes, with zones constructed from 26-connected neighbours of the same gray-level in 3D space (one voxel can be part of only one zone). The entry *(i,j)* of the normalized GLSZM is then defined as:

$$p\left( i, j \right)=\frac{p\left( i, j \right)}{\sum_{i=1}^{N_{g}} \sum_{j=1}^{L_{z}} p\left( i, j \right)}$$

The following quantities are also defined:

$$\mu_{i}=\sum_{i=1}^{N_{g}} i\sum_{j=1}^{L_{z}} p\left( i, j \right)$$

$$\mu_{j}=\sum_{j=1}^{L_{z}} j\sum_{i=1}^{N_{g}} p\left( i, j \right)$$

## SmallAreaEmphasis

$$SmallAreaEmphasis=\sum_{i} \sum_{j} \frac{p\left( i, j \right)}{j^{2}}$$

## LargeAreaEmphasis

$$largeAreaEmphasis=\sum_{i} \sum_{j} j^{2}p\left( i, j \right)$$

## IntensityVariability

$$IntensityVariability=\frac{1}{N_{g}\times L_{z}}\sum_{i} \sum_{j} \left( ip\left( i, j \right)-\mu_{i} \right)^{2}$$

## SizeZoneVariability

$$SizeZoneVariability=\frac{1}{N_{g}\times L_{z}}\sum_{i} \sum_{j} \left( jp\left( i, j \right)-\mu_{j} \right)^{2}$$

## 3.5 ZonePercentage

$$ZonePercentage=\sum_{i} \sum_{j} \frac{\sum_{i} \sum_{j} p\left( i, j \right)}{\sum_{j} j\sum_{i} p\left( i, j \right)}$$

## 3.6 LowIntensityEmphasis

$$LowIntensityEmphasis=\sum_{i} \sum_{j} \frac{p\left( i, j \right)}{i^{2}}$$

## 3.7 HighIntensityEmphasis

$$HighIntensityEmphasis=\sum_{i} \sum_{j} i^{2}p\left( i, j \right)$$

## 3.8 LowIntensitySmallAreaEmphasis

$$LowIntensitySmallAreaEmphasis=\sum_{i} \sum_{j} \frac{p\left( i, j \right)}{i^{2}j^{2}}$$

## 3.9 HighIntensitySmallAreaEmphasis

$$HighIntensitySmallAreaEmphasis=\sum_{i} \sum_{j} \frac{i^{2}p\left( i, j \right)}{j^{2}}$$

3.10 LowIntensityLargeAreaEmphasis

$$LowIntensityLargeAreaEmphasis=\sum_{i} \sum_{j} \frac{j^{2}p\left( i, j \right)}{i^{2}}$$

## 3.11 HighIntensityLargeAreaEmphasis

$$HighIntensityLargeAreaEmphasis=\sum_{i} \sum_{j} i^{2}j^{2}p\left( i, j \right)$$

# **Gaussian Transform**

During image processing, discretization mainly refers to the discretization of the gray value. The method of image filter processing is Gaussian filter processing. The Gaussian filtering principle is employed, and the processing result is the image after noise reduction.

The one-dimensional Gaussian filter function is as follows:

$$G\left( x \right)=\frac{1}{\sqrt{2\pi}\sigma}e^{-\frac{x^{2}}{2\sigma^{2}}}$$

The two-dimensional Gaussian filter function is as follows:

$$G\left( x,y \right)=\frac{1}{2\pi\sigma^{2}}e^{-\frac{x^{2}+y^{2}}{2\sigma^{2}}}$$

The principle of the Gaussian filter is to discretize the Gaussian function and use the Gaussian function value at the discrete point as the weight. A weighted average of a range of neighborhoods is generated for each pixel of the gray matrix, which can effectively eliminate Gaussian noise.
