# Supplementary material for: Radiomics Analysis of Iodine-Based Material Decomposition Images With Dual-Energy Computed Tomography Imaging for Preoperatively Predicting Microsatellite Instability Status in Colorectal Cancer
Source: Front Oncol. 2019 Nov 22;9:1250. doi: 10.3389/fonc.2019.01250 (PMC6883423; doi:10.3389/fonc.2019.01250)
Supplement: Supplementary file 2 [file Table_2.DOCX]

**Supplementary II: The details of loss function value (**$\boldsymbol{L}\left( \boldsymbol{w} \right)$**)**

In our study, the 5-fold cross-validation technique was used for model selection. The data in training set were divided into five subsets equally. Then, four subsets were selected each time to train, and the remaining one subset was used to test. By changing the sub-test set in turn, five loss function values ($L\left( w \right)$) during above five models would be obtained. The average value of $L\left( w \right)$ was calculated. When average $L\left( w \right)$ reached a minimum value, the optimization of the logistic regression model would be completed, and the final model would be constructed.

Supplement II：The details of $L\left( w \right)$ are as follows:

In fact, the 5-fold cross-validation technique has been applied during the LASSO selection process. LASSO algorithm introduces the L1 regular term on the basis of the logarithmic loss function, the parameter $\lambda$ is optimized when the loss function value ($L\left( w \right)$) is minimized. When $L\left( w \right)$ reaches a minimum value, the model reaches an optimal status.

LASSO is a relatively mature variable selection algorithm and is suitable for feature selection of high-dimensional radiomics^[1]^. The LASSO algorithm introduces the L1 regular term through the cross-validation technique, and the regularization technique optimizes the model from the perspective of the loss function value ($L\left( w \right)$) to minimize the model overfitting. The formula of the algorithm is as follows:

$$L\left( w \right)= \frac{1}{m}\sum_{i=1}^{m} \left[ ln\left( 1+exp\left( \boldsymbol{\beta}\cdot x^{\left( i \right)} \right) \right)-y^{\left( i \right)}\left( \boldsymbol{\beta}\cdot x^{\left( i \right)} \right) \right] +\lambda\frac{1}{2}\left\| \boldsymbol{\beta} \right\|_{1}$$

Where$L\left( w \right)$ is the loss function of the model, $m$ is the sample size, $x^{\left( i \right)}$ and $y^{\left( i \right)}$ represent the observation value of the i^th^ sample size and its end point outcome respectively, $\left\| \boldsymbol{\beta} \right\|_{1}$is the penalty of the feature vector $L_{1}$, $\left\| \boldsymbol{\beta} \right\|_{1}= \left| \beta_{1} \right|+\left| \beta_{2} \right|+\ldots+\left| \beta_{p} \right|$.

LASSO algorithm removes the redundancy between the radiomics features from the model level. After the LASSO process is completed, we can extract the remaining features and their corresponding weights. After bringing them back to the formula, this is essentially a logistic regression model. LASSO binary logistic regression has been a widely used combination^[2]^.

The details of optimization of the logistic regression model function are as follows:

$$P\left( y=1|\boldsymbol{x} \right)=\frac{\exp\left( \hat{\boldsymbol{\beta}}\cdot\boldsymbol{x} \right)}{1+\exp\left( \hat{\boldsymbol{\beta}}\cdot\boldsymbol{x} \right)}$$

***=***

- 4.22e-01

- 9.26e-01 × gender.1

+ 3.03e-14 × gender.2

- 3.07e-01 × smoking.1

+ 1.11e-16 × smoking.2

+ 9.18e-01 × family history of cancer.1

- 1.49e-03 × family history of cancer.2

- 5.63e-01 × MaxIntensity

- 5.08e-01 × uniformity

- 1.76e-02 × GLCMEnergy_AllDirection_offset6_SD_Gaussian

- 8.25e-02 × GLCMEnergy_angle90_offset8_Gaussian

- 3.70e-02 × GLCMEntropy_AllDirection_offset8_Gaussian

- 6.30e-01 × HaralickCorrelation_AllDirection_offset8_SD_Gaussian

Reference：

1. Sauerbrei W, Royston P, Binder H. Selection of important variables and determination of functional form for continuous predictors in multivariable model building. *Statistics in medicine* (2007) 26(30):5512-5528. doi: 10.1002/sim.3148

[2] Huang YQ, Liang CH, He L, Tian J, Liang CS, Chen X, te al. Development and Validation of a Radiomics Nomogram for Preoperative Prediction of Lymph Node Metastasis in Colorectal Cancer. *J Clin Oncol.* (2016) 34: 2157-64. doi: 10.1200/JCO.2015.65.9128
